# Resistance to Bipyridyls Mediated by the TtgABC Efflux System in *Pseudomonas putida* KT2440

**DOI:** 10.3389/fmicb.2020.01974

**Published:** 2020-08-18

**Authors:** Tania Henríquez, Nicola Victoria Stein, Heinrich Jung

**Affiliations:** Mikrobiologie, Biozentrum, Ludwig-Maximilians-Universität München, Munich, Germany

**Keywords:** resistance-nodulation-division transporter, ion chelator, metal starvation, bipyridyl, *Pseudomonas*

## Abstract

Resistance-nodulation-division (RND) transporters are involved in antibiotic resistance and have a broad substrate specificity. However, the physiological significance of these efflux pumps is not fully understood. Here, we have investigated the role of the RND system TtgABC in resistance to metal ion chelators in the soil bacterium *Pseudomonas putida* KT2440. We observed that the combined action of an RND inhibitor and the chelator 2,2'-bipyridyl inhibited bacterial growth. In addition, the deletion of *ttgB* made the strain susceptible to 2,2'-bipyridyl and natural bipyridyl derivatives such as caerulomycin A, indicating that TtgABC is required for detoxification of compounds of the bipyridyl family. Searching for the basis of growth inhibition by bipyridyls, we found reduced adenosine triphosphate (ATP) levels in the *ttgB* mutant compared to the wild type. Furthermore, the expression of genes related to iron acquisition and the synthesis of the siderophore pyoverdine were reduced in the mutant compared to the wild type. Investigating the possibility that 2,2'-bipyridyl in the *ttgB* mutant mediates iron accumulation in cells (which would cause the upregulation of genes involved in oxidative stress *via* the Fenton reaction), we measured the expression of genes coding for proteins involved in intracellular iron storage and the response to oxidative stress. However, none of the genes was significantly upregulated. In a further search for a possible link between 2,2'-bipyridyl and the observed phenotypes, we considered the possibility that the ion chelator limits the intracellular availability of metabolically important metal ions. In this context, we found that the addition of copper restores the growth of the *ttgB* mutant and the production of pyoverdine, suggesting a relationship between copper availability and iron acquisition. Taken together, the results suggest that detoxification of metal chelating compounds of the bipyridyl family produced by other bacteria or higher ordered organisms is one of the native functions of the RND efflux pump TtgABC. Without the efflux pump, these compounds may interfere with cell ion homeostasis with adverse effects on cell metabolism, including siderophore production. Finally, our results suggest that TtgABC is involved in resistance to bile salts and deoxycholate.

## Introduction

Multidrug efflux pumps are well-known for their role in resistance to antibiotics and other toxic compounds (Nikaido and Takatsuka, 2009; Alvarez-Ortega et al., 2013). In Gram-negative bacteria, the so-called tripartite efflux pumps of the ATP binding cassette (ABC) and the resistance-nodulation-division (RND) superfamilies contribute to multidrug resistance. These systems typically consist of an outer membrane porin, an inner membrane transporter, and an adaptor protein that connects the first two proteins (Li et al., 2015). Genes encoding these transporters are thought to have evolved over millions of years. However, several RND transporters have redundant functions for antibiotic resistance (Poole, 2004; Fernando and Kumar, 2013), raising the question of which natural tasks these systems perform. Roles discussed beyond extrusion of antibiotics include transport of heavy metal ions, quorum sensing molecules, and yet to be identified toxic metabolic products (Fernando and Kumar, 2013; Anes et al., 2015). In addition, the efflux pumps proved to be important for bacterial virulence (Piddock, 2006) and bacteria-plant interactions (Maggiorani Valecillos et al., 2006).

We are investigating functions of RND efflux pumps of the genus *Pseudomonas*. This genus comprises a group of Gram-negative rod-shaped bacteria that includes more than 140 species (Iglewski, 1996), ranging from human or plant pathogens, such as *Pseudomonas aeruginosa* PAO1, to non-pathogenic strains. They can also be beneficial, such as *Pseudomonas putida* KT2440, a plasmid-free isolate (Bagdasarian et al., 1981) that has the capability to induce systematic resistance against certain plant pathogens (Planchamp et al., 2015) and contributes to phytoremediation (Khan et al., 2014). Since RND transporters are important for resistance to toxic compounds (Poole, 2001; Putrins et al., 2010), it is likely that they play vital roles in the colonization of different niches by *Pseudomonas*. The use of RND inhibitors is an interesting tool to study their functions and relevance in nature. In this context, a previous publication revealed that the combined use of iron chelators and RND inhibitors represents a promising therapeutic intervention in *P. aeruginosa* infections (Liu et al., 2010). The authors describe that the use of the iron chelator 2,2'-bipyridyl (Bip) in combination with the RND pump inhibitor Phe-Arg-β-naphthylamide (PAβN) significantly reduces the growth of *P. aeruginosa* PAO1 in comparison to the effect of the individual compounds. However, the relationship between these molecules and bacterial metabolism is not known. Interestingly, Bip is commonly used to generate iron limitation in experimental setups and is widely applied for the study of siderophore secretion (Poole et al., 1991, 1993a,b; Hoegy et al., 2005; Moon et al., 2008; Horiyama and Nishino, 2014; Becker et al., 2018; Henriquez et al., 2019). Derivatives of this compound, such as collismycin A and caerulomycin A, are also iron chelators found in native environments (Kaur et al., 2015; Kawatani et al., 2016) and are produced by different strains of *Streptomyces* spp. (Funk and Divekar, 1959; Gomi et al., 1994). When cells are exposed to these compounds, these and other iron chelators may have intracellular effects (besides causing iron limitation in the environment) such as sequestering iron from iron^-^ dependent proteins in the periplasm and/or the cytosol (Li et al., 2004). However, little is known about how bacterial cells protect themselves from these compounds in their ecological niches.

In this work, we investigated the role of the RND efflux pump TtgABC in the resistance to Bip and related compounds in *P. putida* KT2440. We observed that the combination of Bip and the RND inhibitor PAβN was detrimental for the growth of *P. putida* KT2440, similarly to what was described for *P. aeruginosa* (Liu et al., 2010). Furthermore, inactivation of the TtgABC system by deletion of the gene encoding the transporter component (TtgB) rendered *P. putida* KT2440 susceptible to Bip even in the absence of the RND inhibitor PAβN. These observations indicated that TtgABC was required for detoxification of Bip (e.g., by transporting it out of the cell). Furthermore, the use of another chelator, caerulomycin A, revealed that TtgABC is involved in the detoxification of Bip derivatives present in native environments. These findings support the idea that extrusion of chelating compounds produced by other bacteria or higher ordered organisms is one of the native functions of TtgABC. Further phenotypic analyses of cells grown in presence of Bip revealed reduced ATP levels of the *ttgB* mutant compared to the wild type and a reduced production of the siderophore pyoverdine. Indeed, genes involved in pyoverdine production and iron acquisition were downregulated in the *ttgB* mutant relative to wild type in presence of Bip. Addition of copper restored growth and pyoverdine production of the mutant strain suggesting a link between the metabolism of iron and copper. Finally, our findings corroborate and extend a role of TtgABC in resistance to bile salts and deoxycholate.

## Materials and Methods

### Bacterial Strains and Culture Media

A complete list of the strains and plasmids used in this study is available in Supplementary Table S1. All strains were cultured in King’s Broth (KB) medium (King et al., 1954) and stored as frozen glycerol stocks. The impact of Bip on growth was tested in KB supplemented with 0.5 mM Bip when appropriate. Plasmids (pUCP18 and pSEVA224) were maintained in *P. putida* cells by addition of 1 mg/ml ampicillin, and plasmid-based gene expression was induced by 1 mM IPTG when indicated. For the analysis of the effects of the RND inhibitor PAβN and Bip, 20 μg/ml and 0.5 mM were used, respectively. To analyze the role of TtgABC in detoxification of Bip derivatives, 0.25–0.5 mM of caerulomycin A were used. For the determination of ATP levels by BacTiter-Glo™ (Promega), Mueller Hinton 2 (cation adjusted) from Sigma was used (as recommended by manufacturer’s instructions). For the analysis of the effects of copper and iron supplementation, 50 or 100 μM CuSO_4_ or FeCl_3_ were added to KB medium with or without 0.5 mM Bip.

### Generation of Plasmids and Mutants

Knockout strain for *ttgB* (pp_1385) was generated by homologous recombination using the pNPTS138-R6KT suicide vector (Lassak et al., 2010). To that end, the upstream and downstream regions of this gene were amplified by PCR. Later, the fragments were digested and ligated into the suicide vector. After checking the insert by sequencing, the plasmid was transformed into *P. putida* KT2440 (first recombination) and grown on cetrimide plates containing 10% sucrose to screen for the second recombination. The final strain was confirmed by PCR and sequencing. For complementation, *ttgB* was amplified and cloned into vector (pUCP-Nde and pSEVA224), and the resulting plasmids were used to transform *P. putida* strains. A complete list of primers used in this study can be found in Supplementary Table S2.

### Colony Morphology Assay

The experiment was performed according to the protocol described by Sakhtah et al. (2016) with some modifications. Briefly, 10 μl of overnight cultures in KB medium were spotted on KB with or without 1.0 mM Bip and incubated at 30°C for 24 h. The colonies were photographed after incubation under normal and UV light. The software ImageJ was used to process the images.

### PVD Measurement in the Supernatant

One milliliter of culture (KB medium) from each flask was taken after 2 h of growth in presence of Bip and centrifuged for 3 min at 15000 rcf. The supernatant was collected and taken for fluorescence measurements using a 96-well plate and a CLARIOstar Plus (BMG LABTECH®; excitation: 400 nm; emission: 455 nm).

### Image Analysis

Bacterial cells grown in presence of Bip for 2 h were washed, sonified, spotted on agar pads (2% agarose), and photographed using Leica DMI 6000B fluorescence microscope equipped with a Leica DFC 365 FX camera and an oil-immersion HCX PL APO CS 100X. Images were taken with phase contrast and cyan filter (excitation: 436 nm; emission: 480 nm). ImageJ and MicrobeJ tools were used for fluorescence quantification and image analysis.

### Growth Curves

Overnight cultures in KB were used to inoculate 35 ml of KB medium supplemented with the agent to be tested. The initial OD at 600 nm was adjusted to ~0.1 and cultures were incubated at 30°C with continuous shaking (180 rpm). A sample was taken every hour for absorbance measurement at OD600. Growth curves using a Tecan reader (Tecan Infinite® M200 pro plate reader) or a CLARIOstar Plus (BMG LABTECH®) were performed in 100 μl of culture per well in a 96-well plate (Corning Costar 96-Well Black).

### Gene Expression Assay

Wild type and *ttgB* strains were grown in KB medium at 30°C with continuous shaking (180 rpm). After 140 min (OD600 ~0.8), 1 mM Bip was added and incubation was continued for another 90 min. Then, the OD600 of the strains was adjusted, and RNA was extracted using NucleoZol (Macherey-Nagel) according to the manufacturer’s instructions. RNA concentration, purity, and integrity were determined by NanoDrop ND-1000 and gel electrophoresis (1% agarose gel). DNase I treatment was performed according to manufacturer’s instructions (#EN0521, Thermo Scientific). One microgram of each RNA sample was used for reverse transcription (High-Capacity cDNA Reverse Transcription Kit from Applied Biosystems). Each complementary DNA (cDNA) sample produced was diluted 1:10 and utilized for real-time PCR using the iQ SYBR Green Supermix (BioRad) in a BioRad CFX96 real-time system (BioRad). The expression of the target genes was normalized against the reference gene (*rpoD*) using the wild type strain as the control group. The sequences of the primers used for this experiment can be found in Supplementary Table S2.

### ATP Measurement

To evaluate the levels of intracellular ATP, the BacTiter-Glo™ Microbial cell viability assay from Promega was used according to the manufacturer’s instructions. Briefly, overnight cultures of the strains in Mueller Hinton 2 (Sigma) were used to inoculate 15 ml of fresh medium with or without 0.5 mM Bip. After 2 h of growth at 30°C with continuous shaking (180 rpm), OD600 was adjusted to 0.3 and 100 μl of each culture were added to a Corning® 96-well plate (white flat bottom). Then, 100 μl of the BacTiter-Glo™ reagent was added to each well, incubated 5 min at room temperature in an orbital shaker, and used to measure luminescence in a Tecan reader (Infinite F500). The settings used for the luminescence measurement were attenuation = automatic; integration time = 1,000 ms; and settle time = 0 ms.

### Susceptibility to Toxic Compounds

Growth in Mueller Hinton (MH) medium was assayed using a Tecan infinite® M200 pro plate reader. Each individual culture was started at an OD600 of 0.1 in 150 μl per well. MH medium was preheated to 37°C before adding bile acids or deoxycholate, respectively. Cells were grown for 20 h at 30°C shaking with orbital amplitude of 2 mm.

### Statistical Analysis

The software GraphPad Prism 7 was used for statistical comparison. Unpaired *t*-test, One-way ANOVA, Dunnett’s multiple comparison test, Kruskal-Wallis, Mann-Whitney, and Bonferroni posttests were performed as appropriate. All experiments were performed a minimum of three times.

## Results

### The Combination of 2,2'-Bipyridyl and the RND Inhibitor Phe-Arg-β-Naphthylamide Reduces *P. putida* KT2440 Growth Due to TtgABC Inhibition

**Figure 1:**
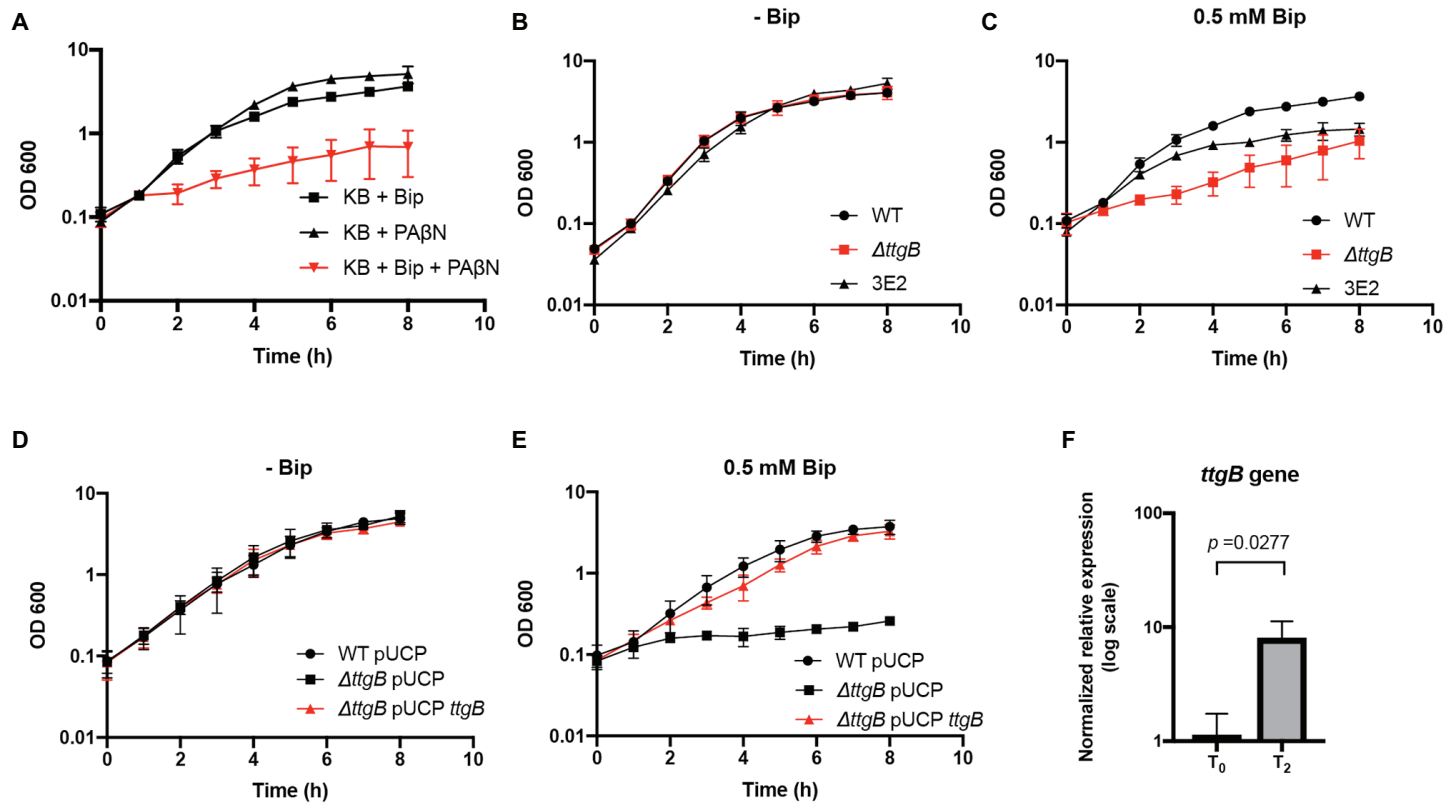
A functional TtgABC is required for bipyridyl (Bip) detoxification in *Pseudonomas putida* KT2440 in presence of Bip. **(A)** Growth of the wild type was analyzed in King’s Broth (KB) medium supplemented with 0.5 mM Bip, 20 μg/ml Phe-Arg-β-naphthylamide (PAβN), or the combination of both. **(B)** Growth was assessed for wild type, *ttgB* mutant and 3E2 strain (non-producer for pyoverdine) in KB medium without supplementation and **(C)** in presence of 0.5 mM Bip. **(D)** Growth of wild type and *ttgB* mutant transformed with pUCP or pUCP-*ttgB* for complementation was recorded in KB medium plus 1 mg/ml ampicillin without supplementation or **(E)** in presence of 0.5 mM Bip and 1 mM IPTG. For **(A–E)**, washed cells from an overnight culture were resuspended in water and used to inoculate 35 ml of KB medium supplemented with the corresponding compound (initial OD600 of 0.1). The flasks were incubated at 30°C with continuous shaking (180 rpm). OD600 was measured at given time points. **(F)** Relative expression levels for *ttgB* in presence of KB plus Bip in comparison to KB without supplementation (wild type strain). For this experiment, after 2 h of growth at 30°C with continuous shaking (T_0_), Bip was added to the medium, and another incubation was performed for 90 min (T_2_). RNA samples were taken at T_0_ and T_2_ and compared by qRT-PCR using *rpoD* as reference gene. The data are presented as an average of three independent experiments.

A previous publication described that the combination of iron chelators, such as Bip, and an RND inhibitor is detrimental for the growth of *P. aeruginosa* PAO1 (Liu et al., 2010). In this context, we analyzed whether a similar phenomenon can be observed for *P. putida* KT2440. To test this, PAβN, a formerly characterized inhibitor of the RND efflux pump MexAB-OprM (homolog of TtgABC) and other RND transporters of *P. aeruginosa* (Lomovskaya et al., 2001), was added to KB medium in presence or absence of Bip, using the strain *P. putida* KT2440 (=wild type) as experimental model. We found that the individual addition of 20 μg/ml PAβN or 0.5 mM Bip to KB medium did not have a significant effect on bacterial growth (Figure 1A; Supplementary Figure S1A). However, when PAβN and Bip were added simultaneously, there was a significant growth reduction (Figure 1A; Supplementary Figure S1A). We wondered whether this phenotype was specifically related to TtgABC [proposed as the main efflux system to cope with antibiotic resistance in *P. putida* (Teran et al., 2003)]. Therefore, we generated a strain with a deletion in the gene coding for the inner membrane component of the system, *ttgB* (PP_1385), and assessed the mutant phenotype in KB without supplementation and in presence of 0.5 mM Bip. To discriminate between the effects of iron limitation in the culture medium established by Bip and a possible intracellular toxicity by Bip, we utilized *P. putida* strain 3E2, which is not able to produce pyoverdine but contains a fully functional TtgABC system (Matthijs et al., 2009). Our results showed about the same growth dynamics in KB without Bip for all three strains, indicating that the activity of the TtgABC system was not important under this condition (Figure 1B; Supplementary Figure S1B). However, when KB medium was supplemented with 0.5 mM Bip, the *ttgB* mutant grew significantly slower than the wild type strain (Figure 1C; Supplementary Figure S1B), and similar to the wild type strain in presence of Bip and PAβN (Figure 1A). Remarkably, 3E2 strain grew faster than the *ttgB* mutant in the presence of Bip (Figure 1C; Supplementary Figure S1B), suggesting that the growth defect of the latter cannot fully be explained by iron limitation in the supernatant. Since, a previous publication reported that Bip is a chelator that can act by depleting intracellular reservoirs of ferrous iron (de Leseleuc et al., 2012), our results led us to the assumption that the TtgABC system of *P. putida* KT2440 was required for Bip detoxification by transporting the ion chelator back to the extracellular environment. Furthermore, to validate the results caused by *ttgB* deletion, a complementation assay was performed. To that end, *ttgB* was cloned into a high copy number plasmid (pUCP-Nde), which was used to transform the *ttgB* mutant strain. In parallel, wild type and *ttgB* mutant were transformed with the empty vector as controls. Our results showed that all three strains grew similarly in KB medium in absence of Bip (Figure 1D). On the other hand, when Bip was added to the medium in presence of IPTG (to induce expression of *ttgB*), the complemented *ttgB* mutant grew similar to the wild type (Figure 1E).

Finally, we evaluated the expression levels of *ttgB* in presence of Bip using *rpoD* (RNA polymerase sigma factor RpoD) as a housekeeping gene. For this experiment, samples were taken after 90 min post Bip addition (see “Materials and Methods” section). Our results showed that *ttgB* is upregulated about 7-fold in the wild type strain in presence of Bip in comparison to KB medium without supplementation (Figure 1F). These results agree with a previous report of the upregulation of MexAB-OprM (homolog of TtgABC in *P. aeruginosa*) in presence of Bip (Poole et al., 1993b).

Taken together, these results indicated that a functional TtgABC system is required for optimum growth of *P. putida* KT2440 in complex medium with Bip.

### TtgABC Is Involved in the Extrusion of Bipyridyl Derivatives

**Figure 2:**
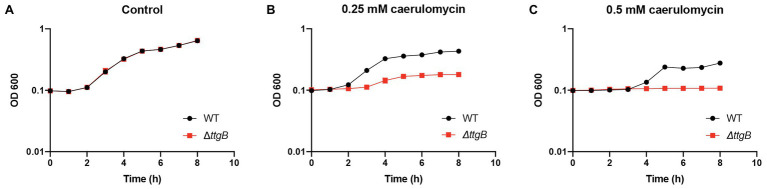
Caerulomycin A inhibits growth when TtgABC is inactive. **(A)** Bacterial growth was analyzed in KB medium without supplementation and **(B)** in presence of 0.25 mM or **(C)** 0.5 mM caerulomycin A. To that end, overnight cultures were used to inoculate 100 μl of KB medium in a 96-well plate. Growth curves were recorded by measuring OD600 using a Tecan reader (Tecan infinite® M200 pro plate reader, settings: 30°C, shaking with an orbital amplitude of 2 mm). All experiments were performed a minimum of three times.

Isomers of bipyridyl can be found in nature and some of them act as mutagenic, cytotoxic or chelating agents (Li et al., 2004). For example, derivatives of the 2,2'-bipyridyl family, such as collismycin A (Gomi et al., 1994) and caerulomycin A, are produced by *Streptomyces* sp., and act as iron chelators in the soil (Funk and Divekar, 1959; Kawatani et al., 2016). We hypothesized that the ability of the TtgABC transporter to extrude Bip and similar compounds came from the necessity of *Pseudomonas* to cope with these molecules in its natural environment. To test this hypothesis, we analyzed the effect of caerulomycin A (Supplementary Figure S2A) on the growth of wild type and *ttgB* mutant. In contrast to KB medium without supplementation (Figure 2A; Supplementary Figure S2B), addition of 0.25 or 0.5 mM caerulomycin (Figures 2B,C; Supplementary Figure S2B) significantly reduced the growth of the *ttgB* mutant in comparison to the wild type strain (Figure 2A; Supplementary Figure S2B). The results are similar to the ones observed with 0.5 mM Bip (Figure 1C). These findings suggest that TtgABC is needed for the extrusion of natural bipyridyl derivatives.

### 2,2'-Bipyridyl Affects the Metabolic State and Pyoverdine Production of the *ttgB* Mutant

**Figure 3:**
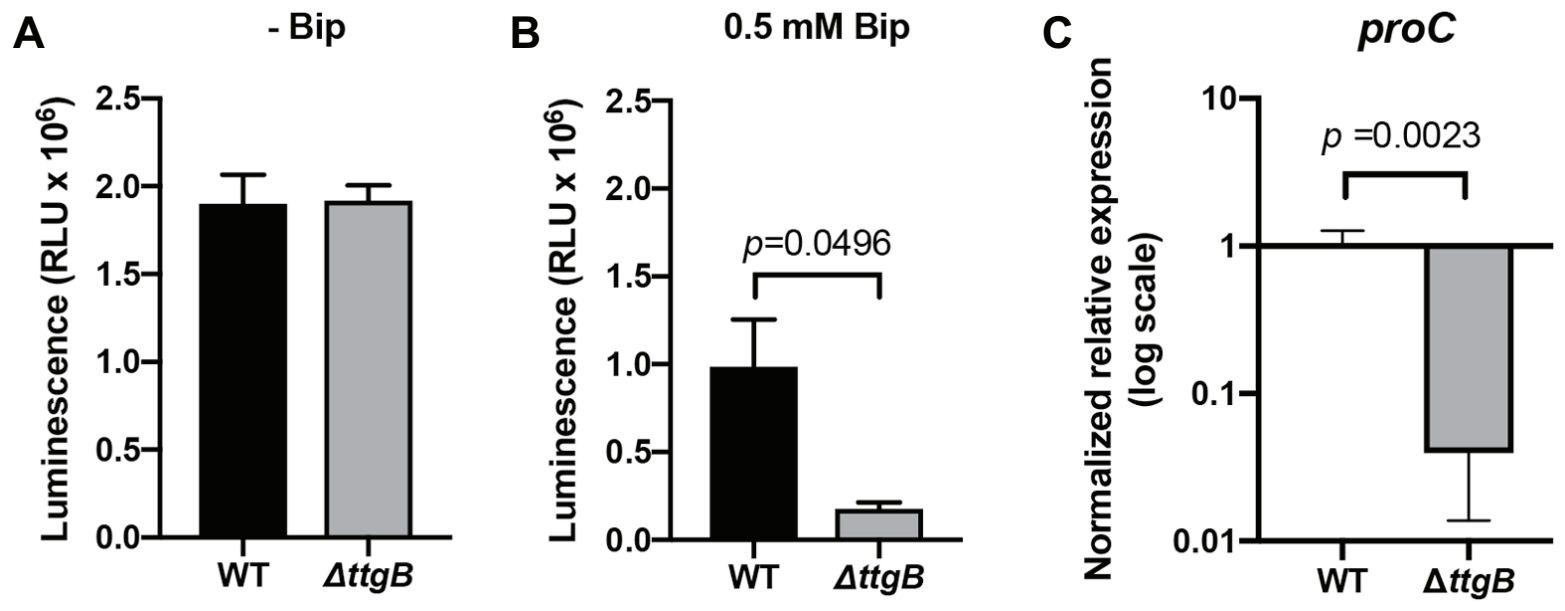
Bipyridyl influences the metabolic state of the *ttgB* mutant. **(A)** Measurements of relative intracellular levels of ATP in cells grown in Mueller Hinton 2 medium without and **(B)** with 0.5 mM Bip. **(C)** Relative expression levels of *proC* in KB plus 0.5 mM Bip for wild type and *ttgB* strain were assessed by qRT-PCR. The target gene was normalized against the reference gene (*rpoD*) using the wild type strain as the control group. The data represent three independent experiments.

To obtain information on the molecular basis of the growth defect observed for the *ttgB* mutant in the presence of bipyridyl derivatives, more detailed phenotypic analyses were performed. A previous publication suggested that the accumulation of small iron chelators inside cells can have severe effects on metabolic pathways, including the production of the energy currency ATP (Santos et al., 2018). In order to test whether this idea applies also to our experimental system, we assessed the impact of 0.5 mM Bip on intracellular ATP levels in wild type and *ttgB* mutant. Our results indicated that in the absence of Bip, both strains contained similar amounts of ATP (Figure 3A). However, in presence of Bip, the *ttgB* mutant showed significantly lower ATP levels than the wild type (Figure 3B). These results support the idea that when the TtgABC system is inactivated, Bip accumulates in cells and negatively affects the bacterial energy metabolism, which consequently impacts cell growth and likely, other metabolic routes. In this context, we also found that the level of expression of *proC*, (coding for pyrroline-5-carboxylate reductase, an enzyme involved in proline biosynthesis), was significantly downregulated (Figure 3C). This result was unexpected, since it is normally used as a reference gene due to its stability (Savli et al., 2003). This result reflects the impairment of the biosynthetic pathways for amino acids and agrees with a low metabolic state.

**Figure 4:**
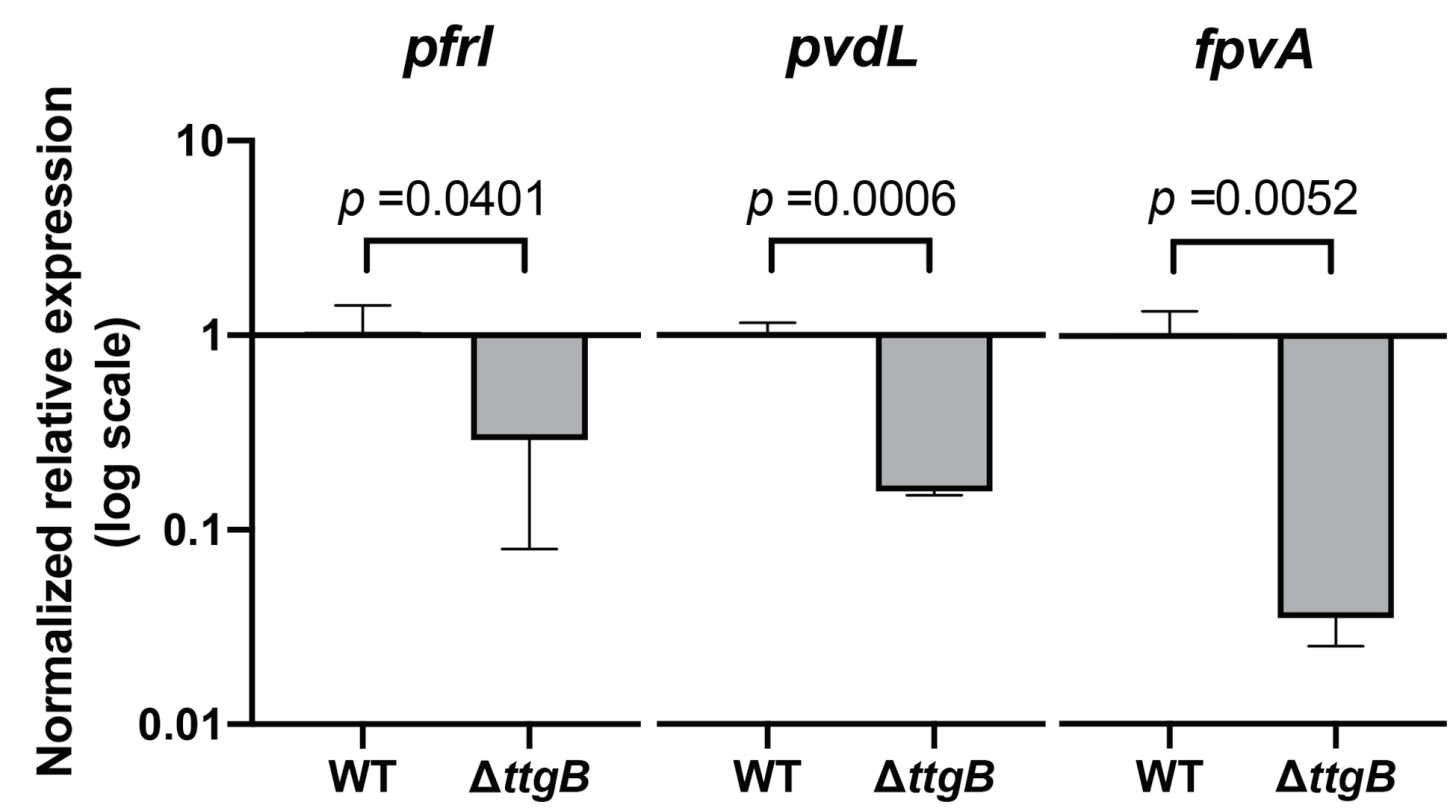
Expression levels of pyoverdine-related genes in the *ttgB* mutant are affected by Bip. Relative expression levels of *pfrI*, *pvdL*, and *fpvA* in KB plus 0.5 mM Bip were assessed by quantitative real time PCR (qRT-PCR). The target genes were normalized against the reference gene (*rpoD*) using the wild type strain as the control group. The data represent three independent experiments.

Next, we investigated the impact of Bip on the synthesis of the siderophore pyoverdine. The idea for this analysis originated from initial phenotypic tests using colony morphology assays. Indeed, we observed that the *ttgB* mutant was unable to produce pyoverdine in a colony grown on KB agar supplemented with 0.5 mM Bip (Supplementary Figure S3A). Similarly, the measurements of pyoverdine in the supernatant (Supplementary Figure S3B) and inside the cells (Supplementary Figure S3C) of *P. putida* in KB plus 0.5 mM Bip, revealed an impaired pyoverdine production by the *ttgB* mutant. This observation was surprising, since Bip is successfully used in many experiments to stimulate production of the siderophore by limiting iron in the medium (Poole et al., 1991, 1993a; Becker et al., 2018; Henriquez et al., 2019). In order to determine whether the expression of pyoverdine synthesis genes in the *ttgB* mutant strain was affected by the presence of Bip, we analyzed the levels of mRNA of genes involved in iron metabolism, including *pfrI* (sigma factor controlling the pyoverdine biosynthetic pathway), *pvdL* (non-ribosomal peptide synthase for pyoverdine production), and *fpvA* (ferripyoverdine receptor). Our results revealed that *pvdL* expression was significantly reduced (about 6.6-fold) in the *ttgB* mutant in comparison to the wild type strain (Figure 4). Similarly, the expression of *pfrI* and *fpvA* was also significantly affected in the mutant in comparison to the wild type strain in the same conditions (Figure 4).

### 2,2'-Bipyridyl Does Not Induce an Oxidative Stress Response

**Figure 5:**
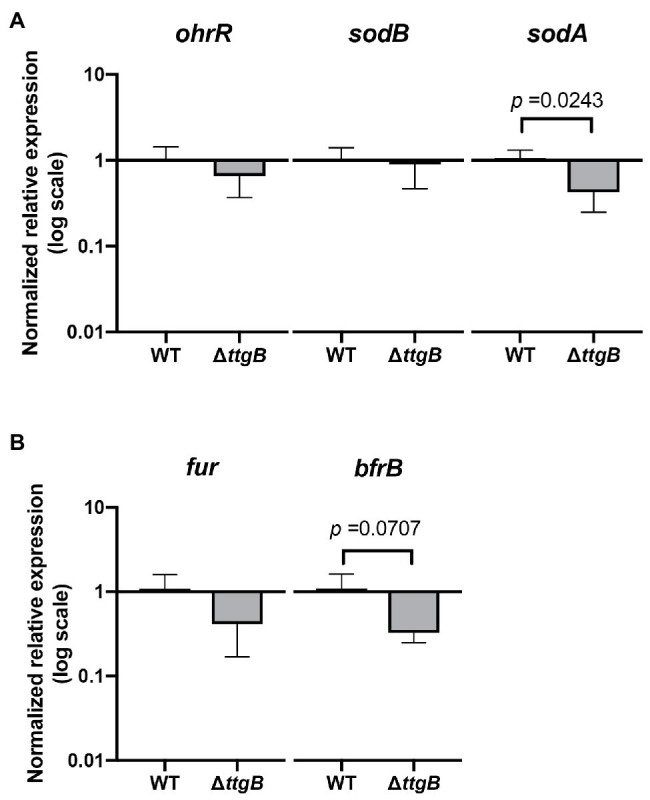
Bipyridyl toxicity is not related to oxidative stress due to iron accumulation. **(A)** Relative expression levels of genes involved in oxidative stress and **(B)** iron metabolism were analyzed by qRT-PCR of mRNA isolated from the wild type and *ttgB* strain in KB plus 0.5 mM Bip. The target genes were normalized against the reference gene (*rpoD*) using the wild type strain as the control group. The data represent three independent experiments.

What is the mechanism of the repression of genes involved in pyoverdine synthesis and iron uptake by Bip? It is well-known that an excess of intracellular iron inhibits transcription of these genes *via* the ferric uptake regulator (Fur; Cornelis et al., 2009). Therefore, we tested the possibility that a non-functional TtgABC system allows the accumulation of iron in cells (due to Bip) leading to oxidative stress *via* the Fenton reaction. For this purpose, we analyzed the expression levels of genes involved in oxidative stress response, such as *ohrR* (encoding the oxidative stress sensor and regulator, OhrR; Atichartpongkul et al., 2010), *sodA* and *sodB* (encoding the superoxide dismutases SodA and SodB; Hassett et al., 1995). mRNA levels of the respective genes were determined by qRT-PCR. The results revealed that that neither *ohrR* nor *sodB* were upregulated by Bip in the *ttgB* mutant (Figure 5A). Contrary to our hypothesis, *sodA* was even significantly downregulated (Figure 5A). These results indicated that Bip does not induce an oxidative stress response in the *ttgB* mutant.

Since elevated intracellular iron concentrations could be compensated by expression of the *bfrB* gene (which encodes the iron storage protein bacterioferritin; Eshelman et al., 2017), we also analyzed its expression. However, *bfrB* turned out to be significantly downregulated (Figure 5B). In the same context, we investigated the expression of *fur*, which was described to be regulated by the levels of iron (De Lorenzo et al., 1988; Carpenter et al., 2009). Our results indicated no significant differences between the wild type and *ttgB* mutant.

Taken together, these results contradict the idea that inactivation of the TtgABC system leads to an intracellular accumulation of iron.

### 2,2'-Bipyridyl Toxicity Is Related to Intracellular Metal Chelation

**Figure 6:**
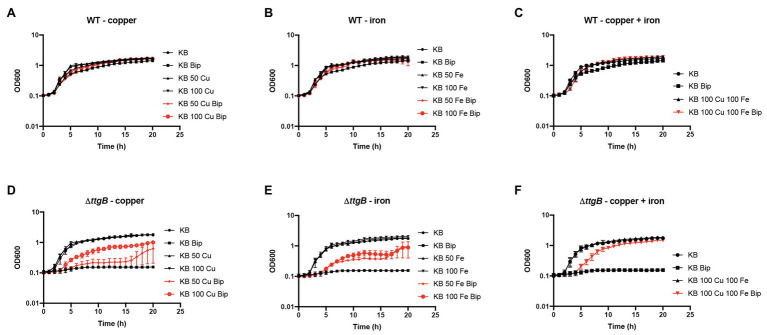
Bipyridyl toxicity in the absence of a functional TtgABC is dependent on metal chelation. Bacterial growth of the wild type strain **(A–C)** and the *ΔttgB* mutant **(D–F)** was assessed in KB medium supplemented with **(A,D)** 50–100 μM CuSO_4_, **(B,E)** 50–100 μM FeCl_3_ or **(C,F)** both metal salts. To that end, washed cells from an overnight culture were resuspended in water and used to inoculate 100 μl of KB medium in a 96-well plate. Growth curves (OD600) were recorded using a CLARIOstar reader. All experiments were performed a minimum of three times.

If there is no excessive accumulation of iron in cells, how can the downregulation of pyoverdine-related genes and the *fpvA* gene be explained? As an alternative, we hypothesized that Bip interferes with the ion homeostasis of cells by chelating intracellular iron ions or other metal ions. In fact, Bip binds not only iron with high affinity (Fe^3+^: Δ*G* = −137 kJ mol^−1^, Fe^2+^: Δ*G* = −69 kJ mol^−1^) but also copper ions (Cu^2+^: Δ*G* = −120 kJ mol^−1^), and when accumulated in cells, it may compete with cellular proteins for these molecules (Santos et al., 2018). In order to test this possibility in our system, we analyzed the impact of the supplementation of KB medium with FeCl_3_ and/or CuSO_4_ on the growth of the wild type and the *ttgB* mutant in the presence and absence of Bip (Figure 6; Supplementary Figure S4). While the addition of FeCl_3_ and/or CuSO_4_ did not significantly affect growth dynamics of the wild type strain, we found that both metals salts significantly improved the growth behavior of the *ttgB* mutant (Figure 6; Supplementary Figure S4). We also observed that the positive effect of CuSO_4_ supplementation on growth was higher than the one from iron, indicating that Bip would interact primarily with this metal. Indeed, the predicted binding affinity for copper is higher than for ferrous iron (Santos et al., 2018). This result was interesting, since a previous report indicated that copper starvation leads to the downregulation of the genes involved in pyoverdine production in *P. aeruginosa*, both in high‐ and low-iron media (Frangipani et al., 2008). In this context, we assessed the effect of copper supplementation on pyoverdine production over time and observed an increase in the fluorescence intensity of the *ttgB* mutant (Supplementary Figure S5).

These results suggested that the intracellular toxic effects of Bip are related to metal chelation reducing not only the availability of intracellular iron but also of copper. Copper starvation may not only explain the observed inhibition of the synthesis of pyoverdine but also the reduced ATP levels in the *ttgB* mutant in the presence of Bip. In fact, the function of central enzymes involved in energy conversion, like cytochrome c oxidase (respiratory chain), needs copper as a cofactor.

### TtgABC Is Involved in the Resistance to Other Toxic Compounds

**Figure 7:**
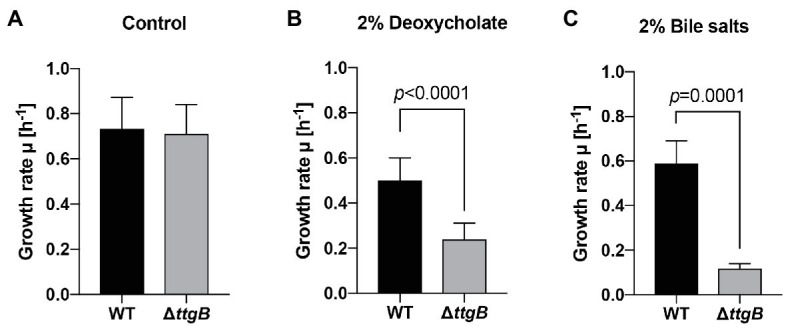
Resistance of *Pseudonomas putida* KT2440 to deoxycholate 2% and bile salts 2% is related to TtgABC activity. **(A)** Growth rates of WT and Δ*ttgB* strain were assessed in Mueller Hinton (MH) medium without addition (control), **(B)** in the presence of 2% deoxycholate and **(C)** in the presence of 2% bile salts. Growth was recorder by measuring OD600 in Tecan reader (Tecan infinite® M200 pro plate reader, settings: 30°C, shaking with orbital amplitude of 2 mm). Growth rates were determined from the exponential growth phase. All experiments were performed a minimum of three times.

Previous publications have already analyzed the role of TtgABC in the extrusion of different compounds (Poole, 2001; Godoy et al., 2010). In order to extend and better characterize this function, we selected 15 potentially toxic chemicals. In this context, we performed susceptibility assays using microdilution method (Supplementary Table S3). Our results showed that the deletion of *ttgB* increased the susceptibility to deoxycholate 2% and bile salts 2% (Figure 7). The expression of *ttgB* from a plasmid complemented the defect in both cases (Supplementary Figure S6). On the other hand, when *ttgB* was deleted, it was not possible to detect a significant effect on the susceptibility toward ZnCl_2_, SDS, Triton X, sodium cyanide, tannic acid, crystal violet, acriflavine HCl, 1-butanol, isobutanol, phenol, rhodamine B, indole, and 3-amino-1,2,4-triazole at the concentrations tested (Supplementary Table S3). Several of these compounds reduced or inhibited growth of all the tested strains (such as Acriflavine and crystal violet in the disc diffusion assay), but did not show a significant difference between the wild type and the *ttgB* mutant (Supplementary Table S3). Altogether, these results strengthen the importance of the presence of TtgABC for the detoxification of toxic compounds.

## Discussion

In this work, we show that the RND efflux pump TtgABC is required for the detoxification of Bip and caerulomycin A, thus protecting the soil bacterium *P. putida* KT2440 from these toxic chelating agents. Evidence for this conclusion is supported by the observation that inactivation of the TtgABC system (by deletion of *ttgB* or the presence of the RND efflux pump inhibitor, PAβN), renders the bacterium sensitive to the chelating agents. Considering that bipyridyl isomers and derivative molecules are widely found in the environment and that they can have mutagenic, cytotoxic, or chelating effects (Li et al., 2004; Kaur et al., 2015; Kawatani et al., 2016), we propose that the extrusion of these compounds by the efflux pump is a trait that is useful for the bacterium in its natural environment. The results are in accordance with the previous observation that an iron chelator combined with an RND efflux pump inhibitor impairs the growth of the pathogen *P. aeruginosa* (Liu et al., 2010). Since a recent publication describes that some compounds present in plants can act as RND inhibitors (Choudhury et al., 2016), it is possible that this interaction occurs in nature and represents an important obstacle for the colonization of respective niches (for example, plant surfaces) by *Pseudomonas* species.

Searching for mechanisms behind the toxicity of bipyridyl compounds in the *ttgB* mutant, we found reduced levels of ATP and a repression of *proC*, a housekeeping gene required for proline biosynthesis and routinely used to normalize gene expression data (Savli et al., 2003). These results suggest that bipyridyls have a more general effect on the cell metabolism, including inhibition of energy converting processes. In addition, we found an inhibition of the production of the siderophore pyoverdine in the *ttgB* mutant. In accordance with this observation, genes required for pyoverdine synthesis (*pvdL*) and iron uptake (*fpvA*), including the gene of the sigma factor PfrI (=PvdS in *P. aeruginosa*), are repressed. The latter result is surprising, since Bip is routinely used to create iron limitation in the environment to stimulate pyoverdine production (Poole et al., 1991, 1993a,b; Hoegy et al., 2005; Moon et al., 2008; Horiyama and Nishino, 2014; Becker et al., 2018).

To mechanistically connect the different phenotypes of the *ttgB* mutant, we had two mutually exclusive hypotheses: (i) in the absence of a functional TtgABC system, Bip mediates the uptake of excessive amounts of iron in cells, thereby repressing genes involved in iron acquisition *via* Fur and causing oxidative stress *via* the Fenton reaction, and (ii) without TtgABC, non-complexed Bip accumulates in the periplasm and/or cytosol of the bacteria and exerts toxic effects through binding of cellular metal ions. Testing hypothesis (i), we did neither find an upregulation of genes involved in an oxidative stress response or of the gene coding for bacterioferritin (known to bind excessive amounts of intracellular iron). These results make hypothesis (i) unlikely. Support for hypothesis (ii) comes from previous investigations showing the chelation of metals from enzymes by Bip (Bardsley et al., 1974). Since, besides Fe^3+^ (and Fe^2+^), also Cu^2+^ belongs to the group of metal ions bound by Bip with high affinity (Santos et al., 2018), we analyzed the impact of both ions on growth in the presence of Bip. Indeed, we found that the addition of one or both ions stimulated growth of the *ttgB* mutant (but not of the wild type), suggesting that Bip not only affects the availability of intracellular iron but also of copper. Competition of Bip with metal dependent enzymes and protein complexes for the ions would explain the observed metabolic effects including the diminished levels of ATP (e.g., *via* inactivation of the iron and copper-dependent cytochrome c oxidase). The results are in accordance with a previous publication showing that small ion chelators, such as Bip, have several metabolic effects, including inhibition of the protein biosynthesis and bacterial cytokinesis (Santos et al., 2018). How can the repression of genes required for iron acquisition be explained in this scenario? Highest effects on growth stimulation were observed by addition of Cu^2+^. Also, Cu^2+^ stimulated the production of pyoverdine by the *ttgB* mutant in the presence of Bip, suggesting a connection between iron and copper metabolism. A close relationship between copper and iron regulation was previously described in *Escherichia coli* (Kershaw et al., 2005) and *P. aeruginosa* (Frangipani et al., 2008). This last report also described the negative effects of copper starvation on pyoverdine production (Frangipani et al., 2008). However, how these two metabolic routes are connected is still unknown. In *P. aeruginosa*, PA2384, a predicted DNA-binding protein, was suggested as the clue for copper and iron regulation (Frangipani et al., 2008). Orthologs of the respective genes occur also in other *Pseudomonas* species, including *P. fluorescens*, *P. syringae*, and *P. putida* (PP_2900). Further experimental evidence is needed in order to clarify its role.

Finally, the involvement of TtgABC in resistance to toxic compounds (such as ampicillin, toluene, chloramphenicol, tetracycline, among others) has been previously tested (Poole, 2001; Godoy et al., 2010). Our results extend the information on the substrate specificity of this efflux pump by showing that TtgABC is required for resistance to bile salts and deoxycholate. This activity is also relevant for the adaptation of *Pseudomonas* species to an environment containing these or similar compounds. The results were consistent with studies in other Gram-negative bacteria, such as *E. coli* and *Salmonella*, in which AcrAB-TolC is involved in bile salt resistance (Rosenberg et al., 2003; Prouty et al., 2004).

Altogether, these results provide new insights into the role of the TtgABC system in *Pseudomonas* species by demonstrating its involvement in the detoxification of bipyridyl and related compounds, which we propose as one of the native functions of this RND transporter.

## Data Availability Statement

The datasets generated for this study are available on request to the corresponding author.

## Author Contributions

HJ, TH, and NS designed experiments and analyzed data. HJ and TH wrote the manuscript. TH and NS performed experiments. All the authors contributed to the article and approved the submitted version.

### Conflict of Interest

The authors declare that the research was conducted in the absence of any commercial or financial relationships that could be construed as a potential conflict of interest.
