# Supplementary material for: Resistance to Bipyridyls Mediated by the TtgABC Efflux System in Pseudomonas putida KT2440
Source: Front Microbiol. 2020 Aug 18;11:1974. doi: 10.3389/fmicb.2020.01974 (PMC7461776; doi:10.3389/fmicb.2020.01974)
Supplement: Supplementary file 1 [file Data_Sheet_1.PDF]

Supplemental Material

# Resistance to bipyridyls mediated by the TtgABC efflux system in *Pseudomonas putida* KT2440

Tania Henríquez[1], Nicola Victoria Stein[1], Heinrich Jung[1]*

[1]Mikrobiologie, Biozentrum, Ludwig-Maximilians-Universität München, Martinsried, Germany

*** Correspondence:**
Prof. Dr. Heinrich Jung.
hjung@lmu.de

**Keywords: RND transporter, ion chelator, metal starvation, bipyridyl, *Pseudomonas***

**This PDF file includes:**

Tables S1 to S3
Figure S1 to S6

**Table S1. List of strains and plasmids used in this study.**

| **Strain** | **Description** | **Source** |
|---|---|---|
| WT | *Pseudomonas putida* KT2440 | (Bagdasarian et al., 1981) |
| 3E2 | PP_4220*::*Tn5, pyoverdine-negative Tn5 mutant of *P. putida*, TcR | (Matthijs et al., 2009) |
| Δ*ttgB* | derived from strain KT2440 by deletion of PP_1385 | This work |
| **Plasmids** | | |
| pUCP (pUCP-NdeI) | pUCP-NdeI ($Amp^R$) shuttle vector | (Cronin and McIntire, 1999) |
| pUCP-*ttgB* | Derived from pUCP by cloning the *ttgB* gene from wild type strain into the multicloning site | This work |
| pSEVA224 | $Km^R$; pSEVA221-derivative with *lacI*$^q$/*Ptrc* expression system | (Silva-Rocha et al., 2013) |
| pSEVA224-*ttgB* | pSEVA224-derivative with PP_1385 cloned into the multicloning site | This work |

## Table S2. List of primers used in this study

| Name | Sequence (5'- 3') | Description |
|---|---|---|
| delttgB_2S | GGTATCGACAAAAAACGCCATTCTG | To generate *ttgB* deletion |
| delttgB_2A | TAGTCAGGGATCAGCGAG | To generate *ttgB* deletion |
| delttgB_1A | TGGCGTTTTTTGTCGATACCGTTGA | To generate *ttgB* deletion |
| delttgB_1S | TCAACCAGGAGAACAAGGT | To generate *ttgB* deletion |
| Check ttgB2F | CTGCCAGGCATGTTCGTG | To check second recombination for *ttgB* deletion; sequencing; check *ttgB* deletion |
| Check ttgB2R | TGCGTCGGCGAGTAAGCA | To check second recombination for *ttgB* deletion; sequencing; check *ttgB* deletion |
| Check ttgB-A | CAGGGACAACAAAGACTTG | To check *ttgB* deletion; sequencing |
| Check ttgB-S | GCCAAAGCGGAGTAAACC | To check *ttgB* deletion; sequencing |
| clon ttgB-F | GGAGTAACATATGTCGAAGTTC | To clone *ttgB* |
| clon ttgB-R | AAAGACTTGGTCATTGCC | To clone *ttgB* |
| Seq ttgC-1 | GTTTCTTCGGTCAGCTTGA | Sequencing primer for ttgABC system |
| Seq ttgB-1 | ACAGGGTGTTGTTGATGT | Sequencing primer for ttgABC system |
| Seq ttgA-1 | ATGGTTTACTCCGCTTTGG | Sequencing primer for ttgABC system |
| Seq ttgB-2 | AGCCTTCCTCGGACATGA | Sequencing primer for ttgABC system |
| Seq ttgB-3 | CGTGCTGGCTATCTTCTG | Sequencing primer for ttgABC system |
| Seq ttgA-4 | GCCAAAGCGGAGTAAACC | Sequencing primer for ttgABC system |
| ttgABC-r | AAAAAAAGCTTGCGATAATCGAACGGAATGT | Primers for *ttgABC* amplification |
| ttgABC-f | AAAAATCTAGACCTGAGTACCACCCAGCAGT | Primers for *ttgABC* amplification |
| pUCP-NdeI-MCS-RII | TCATTAATGCAGCTGGCAC | Primer for sequencing MCS of pUCP-Nco |
| M13 Fw(-41) | GGTTTTCCCAGTCACGAC | Primer for sequencing MCS of pSEVA224 |
| M13uni | TGTAAAACGACGGCCAGT | Universal primer for plasmid pNPTS138-R6KT and pUCP-Nco |
| M13reverse | AACAGCTATGACCATG | Universal primer for plasmid pNPTS138-R6KT and pUCP-Nco |
| PP_0387_fwd | TCAACGACCACCTGCCTG | qPCR primer - *rpoD* "housekeeping gene" |
| PP_0387_rev | GGAGCACTCTCGAATACGTTG | qPCR primer - *rpoD* "housekeeping gene" |
| PP_4244_fwd | GATCGCCGCCCGTATCAC | qPCR primer |
| PP_4244_rev | TGAACGATGAGGTGATCTGCG | qPCR primer |
| PP_4243_fwd_JS | GATGACATCGCCTTCCTGCAG | qPCR primer |
| PP_4243_rev_JS | CTTGGCGGATCAGTTGCTCGTTG | qPCR primer |
| pp_4730 F | GCAGAGGATGTCTACAAGG | qPCR primer |

| | | |
|---|---|---|
| pp_4730 R | AGCTCGAACACTGCATGAC | qPCR primer |
| pp_4217 F | GAGCTACCAGATCCAAGGC | qPCR primer |
| pp_4217 R | CCAAACCCTTACCATCGAT | qPCR primer |
| pp_1860 F | ACAACCAGGTCTGTTTCG | qPCR primer |
| pp_1860 R | GAGCATGGCAAGGTATTG | qPCR primer |
| pp_1082 F | CGTGCTCGAAGATATCCTC | qPCR primer |
| pp_1082 F | ACTGCAGGTAGTTCTCGATACC | qPCR primer |
| ttgB F2 | GTGTTCACGGTCAACGGT | qPCR primer |
| ttgB R2 | ACACGCTGTTTTCCTTCG | qPCR primer |
| sodA F | GGAATTCACTGACATGCCCC | qPCR primer |
| sodA R | GTAAGTCTGGTGATGCTTGG | qPCR primer |
| sodB F | AACACCTATGTCGTGAACCT | qPCR primer |
| sodB R | TTCCAGTAGAAGGTGTGGTT | qPCR primer |

**Table S3. Compounds used for susceptibility assays with no effect on *ttgB* deletion.**

All compounds inhibited growth of *P. putida* KT2440 (wild type) and the derived *ttgB* mutant at the given concentration. There was no significant difference in the growth behavior of wild type and *ttgB* mutant.

| **Compound** | **Tested concentrations** | **Assay** |
|---|---|---|
| 1-Butanol | 0.5 – 2% (v/v) | 96 well plate, disc diffusion |
| 3-Amino-1,2,4-triazol | 5 mM – 20 mM | 96 well, disc diffusion |
| Acriflavine HCl | 100 - 400 µg/ml; disc 250 µg | 96 well plate, disc diffusion |
| Crystal violet | 0.0004 – 0.1% (w/v) | 96 well plate, disc diffusion |
| Indole | 0.4 – 1 mM | 96 well plate |
| Isobutanol | 0.5 – 2% (v/v) | 96 well plate |
| Phenol | 400 - 1000 mg/l | 96 well plate |
| Rhodamine B | 100 – 400 µg/ml; disc 250 µg | 96 well, disc diffusion |
| SDS | 0.4 -1% (w/v) | 96 well, disc diffusion |
| Sodium cyanide | 0.5 – 2 mM | 96 well plate |
| Tannic acid | 1 - 4 mg/ml | 96 well plate |
| Triton X | 0.4 -1% (w/v) | 96 well plate |
| $ZnCl_2$ | 4.25 – 5 mM | 96 well plate |

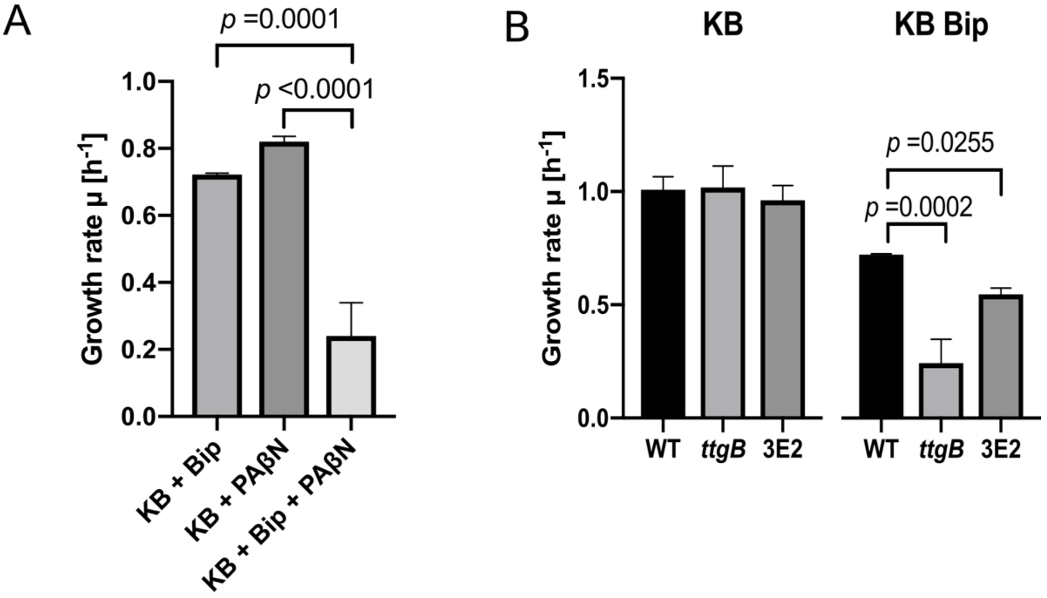


**Figure S1. Effect of different compounds on bacterial growth rate.** (A) Growth of the wild type was analyzed in KB medium supplemented with 0.5 mM Bip, 20 µg/ml PAβN, or the combination of both. (B) Growth was assessed for wild type, *ttgB* mutant and 3E2 strain (non-producer for pyoverdine) in KB medium without supplementation (left) and in presence of 0.5 mM Bip (right). For (A) and (B), information from the exponential phase was used to calculate specific growth rates. One-way ANOVA with Dunnett's multiple comparison test was used. The experiments were performed a minimum of three times.

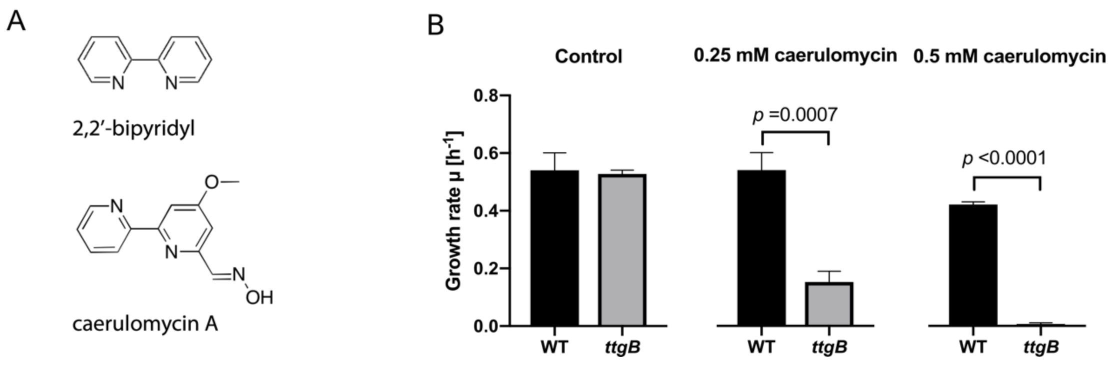


**Figure S2. Effects of caerulomycin A on bacterial growth.** (A) Chemical structures of selected bipyridyls**.** The diagram shows the structure of 2,2'-bipyridyl (top) and one of its natural derivatives, caerulomycin A (bottom). **(**B) Bacterial growth rate in absence or presence of different concentrations of caerulomycin A. Information from the exponential phase was used to calculate specific growth rates. For statistical analysis, the t-test was used. The experiments were performed a minimum of three times.

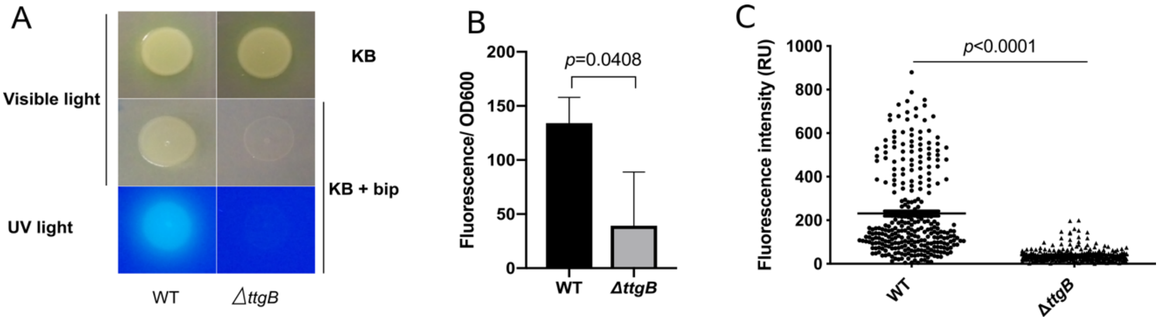


**Figure S3. Pyoverdine production of the *ttgB* mutant in comparison to *P. putida* KT2440 (WT).** (A) Colony morphology assay was performed in KB with or without 1 mM Bip. To that end, 10 µl of an overnight culture were spotted onto each agar plate. After 24 h of incubation at 30°C, the results were visualized under visible and UV light. For image processing and montage creation, the software ImageJ was used. (B) Pyoverdine levels were analyzed in the supernatant (secreted pyoverdine) and (C) inside the cell (pyoverdine accumulation in the periplasm). To that end, after 2 h of growth in KB supplemented with 0.5 mM Bip, 1 ml of culture was taken and centrifuged. The supernatant was used for fluorescence measurement using Tecan reader (Tecan infinite® M200 pro plate reader, excitation: 400 nm; emission: 455 nm) and the cells on the pellet were analyzed and photographed on agarose pads with Leica DMI 6000B microscope. ImageJ and MicrobeJ were used for fluorescence quantification and image analysis. The data are presented as an average of three independent experiments.

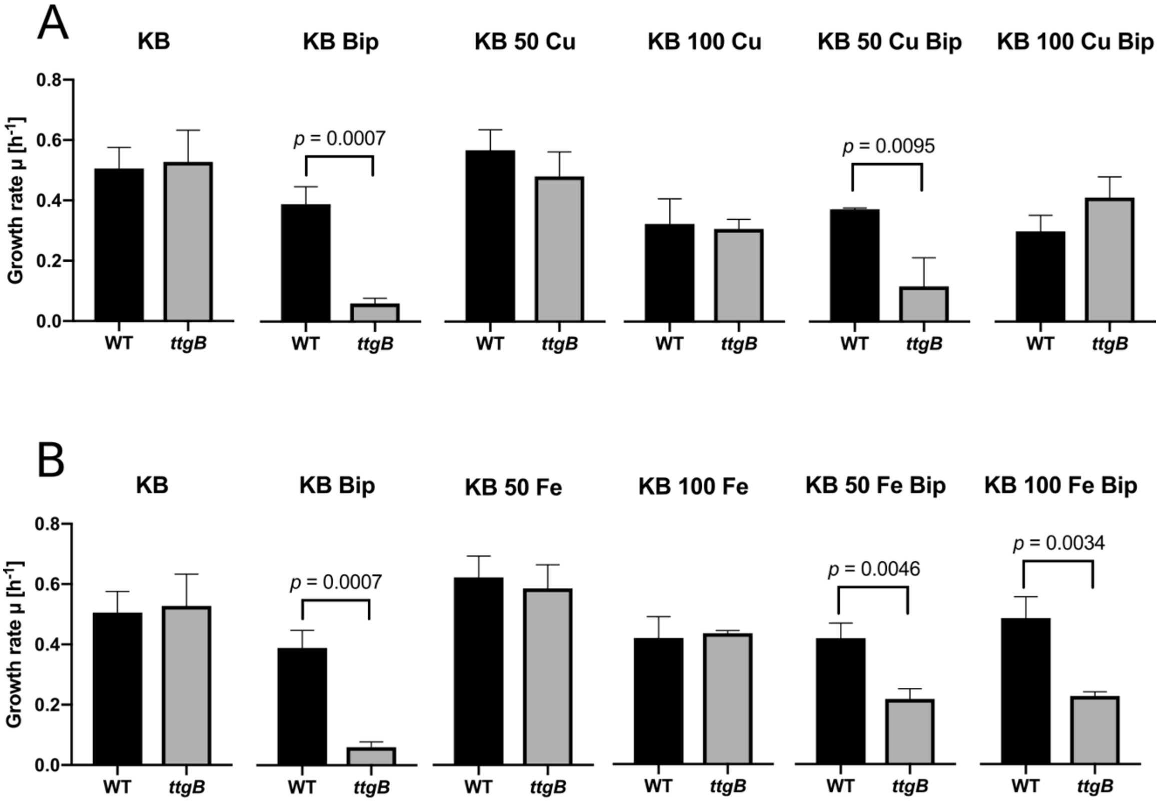


**Figure S4. Effect of copper and iron supplementation on growth rate.** Bacterial growth of the wild type strain and the *ΔttgB* mutant was assessed in KB medium supplemented with (A) 50-100 μM $CuSO_4$ or (B) 50-100 μM $FeCl_3$. Information from the exponential growth phase was used to calculate specific growth rates. For statistical analysis, the t-test was used. The experiments were performed a minimum of three times.

A

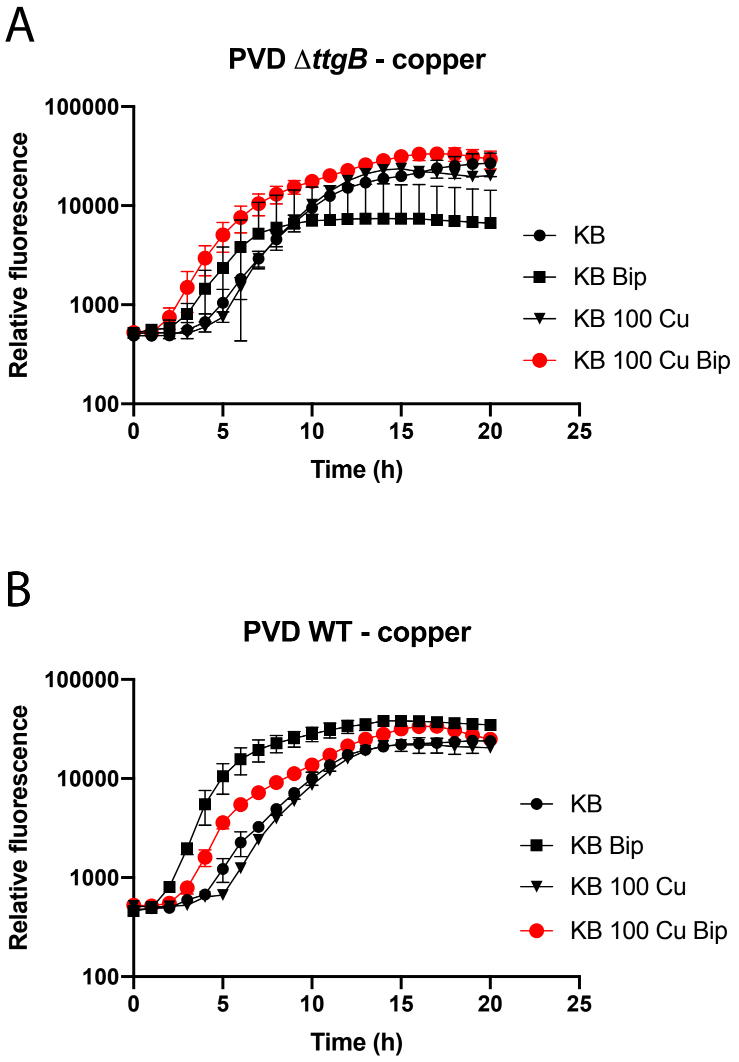


**Figure S5. Effect of copper supplementation on pyoverdine production.** Bacterial growth of *ΔttgB* strain (A) and WT (B) was assessed in KB medium supplemented with 100 µM copper (and 0.5 mM Bip, when appropriate). To that end, washed cells from an overnight culture were resuspended in water and used to inoculate 100 µl of KB medium in a 96-well plate. Pyoverdine fluorescence was detected using a CLARIOstar reader with the following settings: excitation: 400 nm; emission: 455 nm. All experiments were performed a minimum of three times.

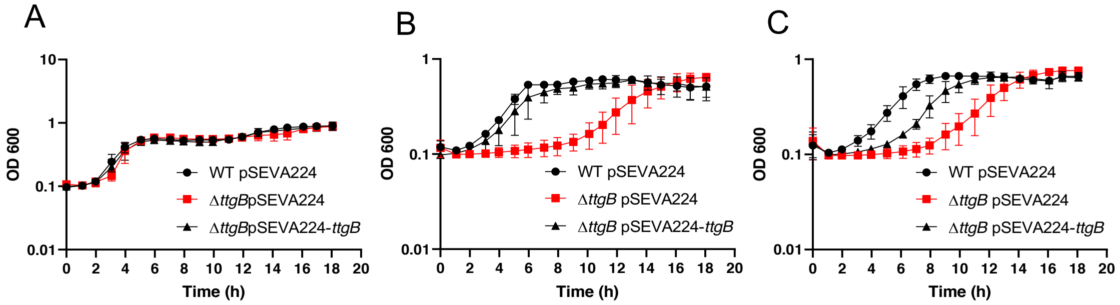


**Figure S6. Resistance of *P. putida* KT2440 to deoxycholate 2% and bile salts 2% is related to TtgABC activity.** (A) Growth of WT, Δ*ttgB* and complemented strain was assessed in Mueller Hinton (MH) medium. Susceptibility testing of strains against (B) deoxycholate 2% and (C) bile salts 2% was performed in 96-well plates. For (A) – (C), Plates were incubated for 18 h at 30°C in a Tecan reader (Tecan infinite®M200 pro plate reader) with shaking (orbital amplitude of 2 mm). All experiments were performed a minimum of three times.

## References


Bagdasarian, M., Lurz, R., Rückert, B., Franklin, F.C.H., Bagdasarian, M.M., Frey, J., and Timmis, K.N. (1981). Specific-purpose plasmid cloning vectors II. Broad host range, high copy number, RSF 1010-derived vectors, and a host-vector system for gene cloning in P*seudomonas*. *Gene* 16**,** 237-247.

Cronin, C.N., and Mcintire, W.S. (1999). pUCP-Nco and pUCP-Nde: *Escherichia-Pseudomonas* shuttle vectors for recombinant protein expression in *Pseudomonas*. *Anal Biochem* 272**,** 112-115.

Matthijs, S., Laus, G., Meyer, J., Abbaspour-Tehrani, K., Schafer, M., Budzikiewicz, H., and Cornelis, P. (2009). Siderophore-mediated iron acquisition in the entomopathogenic bacterium *Pseudomonas entomophila* L48 and its close relative *Pseudomonas putida* KT2440. *Biometals* 22**,** 951-964.

Silva-Rocha, R., Martinez-Garcia, E., Calles, B., Chavarria, M., Arce-Rodriguez, A., De Las Heras, A., Paez-Espino, A.D., Durante-Rodriguez, G., Kim, J., Nikel, P.I., Platero, R., and De Lorenzo, V. (2013). The Standard European Vector Architecture (SEVA): a coherent platform for the analysis and deployment of complex prokaryotic phenotypes. *Nucleic Acids Res* 41**,** D666-675.
